# Serum sodium as a diabetes risk factor?

**DOI:** 10.1111/1753-0407.13365

**Published:** 2023-02-16

**Authors:** Christian W. Mende, Zachary T. Bloomgarden

**Affiliations:** ^1^ Department of Nephrology University of California San Diego California USA; ^2^ Department of Medicine Mount Sinai School of Medicine New York New York USA

Electrolytes play a key role in controlling fluid status, muscle contraction, neurological function, coagulation, and acid base status, as well as multiple enzymatic reactions. Diabetes is associated with multiple significant electrolyte disorders, predominantly affecting serum sodium (Na), potassium, and magnesium.^1^

In healthy individuals, serum Na levels range between 135–146 mmol/L. Hyponatremia is defined as a serum Na < 135 mmol/L, typically accompanied by a low serum osmolality (<275 mosm/L). It is the most frequently seen electrolyte disorder, present in about 5% of adults and 35% of hospitalized patients.^2^, ^3^ In a New York health system analysis of 10 385 patients hospitalized with COVID‐19 in 2020, hyponatremia was present in 37.5% of patients.^4^

## 1 TWO RECENT PUBLICATIONS SUGGEST RELATIONSHIPS BETWEEN SERUM NA AND OUTCOME

1. An article in the *Journal of Diabetes* noted an inverse association of serum Na with new‐onset diabetes among hypertensive patients.^5^ In this study, comparing Na levels from the lowest quartile of 136.7 to the highest quartile of 145.5 mmol/L the group with lower Na had a greater likelihood of new‐onset of type 2 diabetes. This observational study included 4438 hypertensive patients with a mean age of 58.6 years followed for about 3 years. After controlling for variables including body mass index (BMI), gender, baseline blood sugar, estimated glomerular filtration rate, and antihypertensive medications, the authors observed that 12% of those in the third and highest serum Na quartiles, 15% of those in the second quartile, and 17% of those in the lowest Na quartile developed diabetes.
2. A *Lancet* publication analyzed data from 11 255 people enrolled at ages 45–66 in the Atherosclerosis Risk in Community study over a 25‐year follow‐up period, excluding those receiving blood pressure or lipid medications, with fasting glucose>140 mg/dL, known water/salt balance issues, or BMI >35. These authors found serum Na>142 mmol/L to be associated with an 39% increased risk for chronic disease, including diabetes (although the study did not separately analyze diabetes risk), and found that Na >144 mmol/L was associated with 21% increased mortality risk. However, serum Na <138 mmol/L also was associated with higher risk for chronic disease, with “ideal” Na between 138–142 mmol/L. Another observational study based on the US National Health and Nutrition Examination Survey carried out from 2009–2012 also noted increased all‐cause and chronic disease mortality to be associated with serum Na >144 mmol/L.^6^

Low serum Na may activate the renin‐angiotensin‐aldosterone system, may cause increased sympathetic nervous system tone, and may be associated with oxidative stress. Insulin, either administered therapeutically or in the setting of hyperinsulinemia from insulin resistance, increases renal tubular water reabsorption via a presumed vasopressin effect and can result in hyponatremia. Chronic kidney disease and diuretic use in hypertension further increase the risk of hyponatremia as well as other electrolyte imbalances such as hypokalemia and hypomagnesemia.^7^ Serum Na simply may represent the state of hydration. Might some of these mechanisms contribute to effects of even small deviations in serum Na from normal? Should we look more carefully at Na as a biomarker of outcome? Further analysis of large clinical data sets may give answers to these questions.

## References

[1] Liamis G , Liberopoulos E , Barkas F , Elisaf M . Diabetes mellitus and electrolyte disorders. World J Clin Cases. 2014;2:488‐496. 25325058 10.12998/wjcc.v2.i10.488 PMC4198400

[2] Adrogué HJ , Tucker BM , Madias NE . Diagnosis and management of hyponatremia: a review. JAMA. 2022;328:280‐291. 35852524 10.1001/jama.2022.11176

[3] Schmidt BM . The most frequent electrolyte disorders in the emergency department: what must be done immediately? Internist (Berlin). 2015;56:753‐759. 26036654 10.1007/s00108-015-3670-7

[4] Electrolyte abnormalities in patients hospitalized with COVID‐19 , Malieckal DA , Uppal NN , Ng JH , Jhaveri KD , Hirsch JS . Northwell Nephrology COVID‐19 Research Consortium. Clin Kidney J. 2021;14:1704‐1707. 34079619 10.1093/ckj/sfab060 PMC7989524

[5] Cheng Q , Liu X , Cai A , Zhou D , Huang Y , Feng Y . Serum sodium level is inversely associated with new‐onset diabetes in hypertensive patients. J Diabetes. 2022;14:831‐839. 36470584 10.1111/1753-0407.13338 PMC9789394

[6] Stookey JD , Kavouras SΑ , Suh H , Lang F . Underhydration is associated with obesity, chronic diseases, and death within 3 to 6 years in the U.S. population aged 51‐70 years. Nutrients. 2020;12:905. 32224908 10.3390/nu12040905 PMC7230456

[7] Palmer BF , Clegg DJ . Electrolyte and Acid‐Base disturbances in patients with diabetes mellitus. N Engl J Med. 2015;373:548‐559. 26244308 10.1056/NEJMra1503102

